# Being Through Doing: The Self-Immolation of an Asylum Seeker in Switzerland

**DOI:** 10.3389/fpsyt.2018.00110

**Published:** 2018-04-09

**Authors:** Gail Womersley, Laure Kloetzer

**Affiliations:** University of Neuchâtel, Neuchâtel, Switzerland

**Keywords:** trauma, social recognition, immigrants, self-immolation, dialogical analysis

## Abstract

In April 2016, Armin,^1^ an asylum seeker in a village of Switzerland, set himself alight in the public square of the town, one of a few cases reported across Europe. He performed the act following a denied request for asylum and was saved by bystanders. We present the results of two qualitative interviews conducted with Armin, his translator and his roommate following the incident. The act is theorized through the lens of a dialogical analysis focusing on the concept of social recognition. The notion of trauma is considered as a key mediating mechanism, theorized as creating ruptures in time, memory, language, and social connections to an Other. We conclude this communicative act to represent both “being-toward-death” and a relational striving toward life; a “destruction as the cause of coming into being.”

## Introduction

The number of refugees seeking asylum across the world is unprecedented. According to the UNHCR,^2^ 65.6 million people around the world have been forced from home. Among them are nearly 22.5 million refugees—people fleeing conflict or persecution who are defined and protected in international law, and must not be expelled or returned to situations where their life and freedom are at risk. Switzerland has 40,900 legally recognized refugees^3^ and has received over 18,000 more requests for asylum in 2017 alone.^4^ Not least among the difficulties are public health challenges of the multiple traumas faced by this population which constitute severe threats to human, social, cultural, and community development (1–3). Several scholars have demonstrated the ways in which refugees and asylum seekers have attained visibility in protests as well as through other acts of political activism through the practices of hunger strikes, self-mutilation, and lip sewing (4–7). For such vulnerable populations, these acts of self-harm have been theorized as a resilient attempt to overcome invisibility. When there are no words, when oppressed and dispossessed minorities find themselves on the outskirts of public visibility, one recourse is to use the body as a communicative tool. One extreme example of this is self-immolation, used as a tool by various oppressed groups, notably including asylum seekers and refugees, around the world (8–11). A large body of literature further highlights the mediating effect of trauma and dissociation on acts of self-harm [for a comprehensive review, see Ref. (12)]. The high prevalence of self-harm among asylum seekers may therefore come as no surprise. As noted by Finklestein and Solomon (13) and others, there has been an increasing awareness of the traumotogenic nature of the refugee experience. This includes a focus on the “systemic trauma” (14) related to the “experiences of systematic oppression, loss, displacement, and exposure to violence” (15) faced by displaced populations. There is therefore a “double” rupture—one related to traumatic events experienced in the country of origin, the second related to issues of displacement and social isolation experienced by displaced populations.

This “double rupture” invokes a vicious cycle of trauma and isolation, a series of disruptions to the relational processes in which meaning is dialogically created—the bedrock of which is social recognition. Exposure to trauma, itself connected to a breakdown in social connection, risks the individual being caught up in a vicious cycle where no addressee may be found, no language exists to form a coherent narrative whereby the event may itself be collectively made sense of. Viewed through a dialogical systems lens, the traumatic world’s slipping away from the categories of meaning can be seen as a severe disruption of those relational processes in which meaning is formed (16). Thus, as illustrated in the model below, trauma begets trauma.
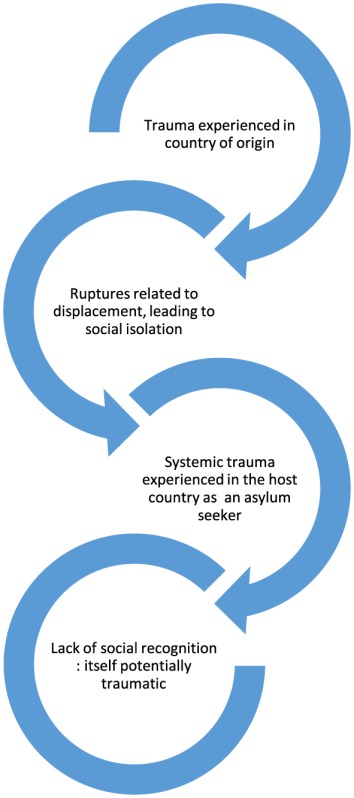


For those whose landscapes have been disrupted by trauma, “their problem is not the limits of memory but of language—the inadequacy of ordinary words to express all they have witnessed” (17). When words fail, when “the temporality of linguistic convention, considered as ritual, exceeds the instances of its utterance, and that excess is not fully capturable or identifiable” (18), it naturally falls upon the body to become the site of (reconstructive) action. Butler, for example, has marked the body as the stage on which traumatic disconnection unfolds:

Loss and vulnerability seem to follow from our being socially constituted bodies, attached to others, at risk of losing those attachments, exposed to others, at risk of violence by virtue of that exposure … the body implies mortality, vulnerability (19).

When the image or content of a traumatic memory is unavailable, it is the bodily aspects of memory which persist. In the absence of language, the body holds what the mind cannot.

## Background

In this article, we explore the self-immolation of Armin, an asylum seeker from North Africa who set himself alight in a public square in a town in Switzerland. We will argue that his experience of being a failed asylum seeker, characterized by a social and political marginalization, as well as the fact of being torn from his communal and social fabric in Africa, could be conceptualized as a traumatic experience. Here, to understand what may or may not be defined as trauma, we draw upon psychoanalytic conceptualizations of the term: trauma is defined as a frightful experience which overwhelms the psyche to such an extent that images, words, or other memories related to the event are unable to be integrated into the system of representations which structure the experience of the individual. Within this paradigm, one commonality of trauma experience is the feeling of a chaos of seemingly unutterable experiences collapsing into that “wordless nothing” (20). The act of self-immolation in particular is understood to reflect this disintegration. It will be explored through the lens of the theoretical construct of “social recognition”—and thus considered as a dialogical utterance used in an attempt to restore intersubjectivity and heal from trauma.

## Social Recognition in Dialogism

Dialogically speaking, an utterance can never stand on its own: it is always directed at the Other. It requires and anticipates a response from the Other (21).

According to Bakhtin (22), “addressivity” is essential to language, it is a lived social reality, continually and creatively co-constructed in micro-interactions and inevitably socially, politically, and historically contextualized. It is intersubjective. Bakhtin’s notion of utterance recognizes that the speaker is already taking cognizance in some way of their addressee, their imagined audience, and thus, the utterance is “dialogic.” Social recognition may therefore be understand as a key component of restoring intersubjectivity:

There is a certain kind of speech that is necessary for [the] actualization of the person to take place; that addresses him directly … language binds them both together, and she is part of a human plurality with him (20).

Marková (21) examines the inherently social nature of dialogism, drawing on the concept of social recognition *(anerkennung)* first introduced by the philosopher Fichte. Within this paradigm, the integrity of the Self is seen to arise in and through the Self’s ethical obligations with respect to the Other: “in dialogical interaction there is no possibility of the Self’s escape from responding to the Other. Even no response is a response” (p. 110). Within this dialogic framework, the Self requires the social recognition of the Other to be fully constituted as a free being. Without a “bridge back to the quotidian,” through social recognition, the symbolic capacities for articulating and validating within a shared dialogical space between Self and Other diminishes and “the publically interpreted world collapses … into non-relationality (unsharability)” (23). In such a “non-dialogic space” (16), the lack of social recognition to connect the individual to a stable external reality may result in experiences of extreme self-loss and, potentially, “the disintegration of the world” (24). As stated by Kirmayer (17):

The mode of remembering and telling one’s story changes greatly when one does not have the poet’s task of evoking in the listener anything approximating the horror that one has experienced …. There is a need to have the rupture with ordinary experience acknowledged by others (p. 25).

Could the act of self-immolation, then, be a consequence of such a disintegration? An attempt to restore intersubjectivity in the face of a loss of social recognition? Both a manifestation of trauma and an attempt to heal from it? To explore this, we turn to the case study of self-immolation by the asylum-seeker Armin, which took place in Switzerland in 2016. Our findings are based on two personal interviews with him following the incident, interviews with his roommate and his translator as well as his personal folder including documentation of his consultation with doctors and lawyers which he himself put together and shared with us. Written informed consent was obtained from the participant for the publication of this case report. This investigation did not fall within the auspices of a particular research project but reflects our interests as academics and, above all, human beings who found ourselves particularly struck by the incident. The theoretical framework guiding our analysis is that of dialogism (21, 25), with a particular focus on self-recognition. We conducted a dialogical analysis of the transcripts of interviews we conducted with Armin as they unfolded, where the focus was on exploring the use of elements drawn from the cultural-symbolic field to confer meaning to past traumatic experiences, his act of self-immolation, the current situation, and to redefine–reposition himself toward the future.

## The Self-Immolation of Armin

Armin is an asylum seeker from North Africa who arrived in Switzerland in September 2014. He had been imprisoned in his country of origin in 2008 after having physically attacked a judge in an attempt to gage out his eyes. This incident occurred during a court case involving a land dispute wherein the judge ruled against his favor. In prison, he began engaging in hunger strikes to protest against the conditions. After serving 6 years out of a 20-year sentence, he managed to escape and make his way to Europe.

Upon his arrival in Switzerland, he was transferred to multiple reception centers where he would go on hunger strike for 8 days at a time to protest against the reception conditions. He describes, “sleeping on the sofa with eight people living and breathing in the same room.” The situation becomes unbearable for him to such an extent that he began sleeping in the bathroom. He was subsequently transferred to a private apartment which he shared with an Eritrean refugee. He began using marijuana and cocaine “because it helped me to forget my problems,” and was arrested by the police for shoplifting while intoxicated. He also started grinding up paracetamol and selling it as cocaine—using the money to send back to his mother. In November 2015, he heard news of his 11-year-old daughter drowning to death in his country of origin.

After having waited for 23 months for the results of his second asylum application, he attempted to leave Switzerland by asking to annul his asylum application. He boarded a train heading for Germany. However, due to the fact that his fingerprints were already registered, he was prevented from leaving the country. In November 2015, he heard news of his sole remaining child, his 12-year-old son, being killed. It was during the same time that he heard of the fact that his application for asylum had been refused, a decision which he decided to appeal. According to his personal file, he attempted suicide in February 2016 following an argument with his doctor and was sent to a psychiatric hospital. Upon returning to his apartment, he received the second negative response to his request for asylum, with a deadline to leave Switzerland by the 7th of July.

On April 20th, the morning of the self-immolation, his roommate reports that the police came to his apartment at 5 o’clock in the morning looking for drugs. According to his roommate, he was detained at the police station until 11:00 a.m., whereupon he was released and returned home for lunch. His roommate describes his mood as being “calm and quiet” during the meal. Thereafter, he headed to the town’s biggest public square with a can of petrol in his bag. In his words, “I was very very angry and very … I poured the petrol onto myself and the people stopped me … I found that there was no other solution but death and that’s why I took the petrol and a lighter and set myself alight.” According to newspaper reports, passers-by heard him screaming incoherently in Arabic and rushed to pour water over him to extinguish the flames.

The incident was poorly reported in the media and few if any public statements were made on behalf of any of the institutions working with asylum seekers. The little that was reported focused mainly on applauding the quick-thinking actions of the citizens and local fire brigade in putting out the fire. One newspaper report concluded that the act did not seem to have been of a political nature. Little was said by the community of asylum seekers and refugees themselves living in the town. The following day, roughly 70 people congregated in a demonstration in solidarity. There was no report of this demonstration in the local media. It was as though a veil of silence shrouded the incident. Armin ended up in intensive care for a month with severe burn injuries, and 7 weeks later was subsequently transferred to a psychiatric hospital.

## Analysis

The first interview we conducted with him was at this psychiatric hospital, with the assistance of a translator. Upon meeting him, the first words he said were, “We find ourselves in a country where we are considered terrorists … we need to unite.” He stated that he planned to go to Geneva the following day to visit the head office of Al Jazeera, the international news network. He wanted to be interviewed to tell people that “the prison of my people in Africa is better than the prison here … I want to explain to my people what Switzerland actually is.” From the first interview, what Armin immediately highlights is wanting to “show” and “explain” to people the dire reality of his situation, for his suffering to be seen. It is the need for social recognition.

He went on to describe a life without meaning: an endless and empty repetition, a wordless nothing:

There’s nothing to do in the apartment. You eat, you drink, you sleep, you eat, you drink, you sleep, you eat, you drink, you sleep. There is nothing.

There appears to be a striking absence of connection to Others mentioned in his speech. The endlessness is repetitive; past and future are circular, not linear. He continues the conversation by reflecting on his initial arrival to Switzerland, where he was housed in a reception center for asylum seekers staying:

For 8 months sleeping on the sofa and not on my bed where 8 people lived and breathed in the same room.

Armin found the physical proximity to the other living, breathing people unbearable, resulting in him leaving the communal room and isolating himself. He continued the interview by explaining this:

Armin: I slept in the toilets. I slept in the toilets! 15 nights I slept in the toilets!

Translator: Why?

Armin: I took my mattress and went to sleep in the toilets!

Translator: Why?

[Silence]

His silence is striking. There are no words to describe this disconnection, this descent into nothingness. He explained that the center for him had been

An open prison. It’s the place where you return to no matter in which direction you go. So long as there are no offers of work, no internship for mutual benefit, there is no future here.

Once again, time is represented in a circular fashion, not a linear one. No matter the direction, there is a return to this same place of nothingness. Any hope for a future is linked to “mutual benefits”—in other words, a dialogue with an Other who could serve to recognize him, to assist him in creating future plans. In the absence of this Other, Armin remains disconnected not only from society but from his own future; “there are inexplicable events, life is unendurable, and … justice is a mirage” [(p. 108) as cited in Ref. (26)].

He continued

I set myself on fire because I didn’t have any will to live. I found myself in a situation without future, without anything and therefore a worthless person, like this cup … without future, without anything, like this cup. I am like this cup. No future for me, no work, no marriage, no learning.

In this discussion, Armin asserts that there is no future here for him, which he relates to the lack of work or professional training. This seems to be for him both an “entry ticket” to normal life, and a way to overcome suffering. It is not only the economic security of a job which he has lost, but the social security of connection to Others and the recognition of himself as a valued member of society—without which he is “worthless,” something less than human.

He believes there to be no possible life for himself in Europe, there is “no work,” “no school,” “no marriage,” and “no future.” He states the reason for having committed suicide as being “I need to have work, to do something.” Elsewhere, it has been argued that the process of forced migration risks creating an “a-temporal space,” a transitional and disconnected period wherein experiences, skills, connections acquired and built in the past are rendered inaccessible (27), as are any clear perspectives on the future. A consequence of such overwhelming episodes is that the experience seems dissociated—isolated in one’s consciousness (28).

Poignantly, he concludes this interview by saying:

Yes, I went to death.^5^

The second interview, similarly conducted in the presence of a translator, occurred once Armin had been released from the psychiatric hospital. He began by saying:

I came to Switzerland, but Switzerland wasn’t my destination. In my mind, there were other destinations like France, Italy, England, but I was stopped.

His words imply a sense of being stuck, of thwarted dreams and ambitions for the future. He is overwhelmed by the reality of the situation, in rude contrast to what he imagined for his life:

Armin’s words (spoken by his interpreter): After 9 days, I went back home but I encountered the same problems of solitude, there is nothing to do, there is no solution but death. I thought there was no solution but death, and for this reason, I brought petrol and a lighter and I set myself fire.

Interpreter: Why did you choose that way?

Armin: So that people know. I did it so that people could be made aware, to stop despising people like me, to know that all people are equal.

And so far, nothing happens. I stay home, I … I eat, I drink, I sleep. There is nothing. There is no future in Switzerland. No future. I got married, my wife died, my two children died. There is nothing (Translation and exchange in Arabic).

Interpreter: I told him that we were here with him. He told me there is nothing. The dog here is better treated than me.

Armin: In this case, is it not better to die?

Further on in the interview, he continued:

I’m not happy with my life, I do this and that, I do some terrible things, I sell drugs, I take cannabis, things like that and I’m unable to find a solution—not for me nor for my family … I want to live like other people. I don’t consider what I have to be a life. I am not living. (…) I am a shirt and walking trousers, not a human being.

An underlying sentiment of shame pervades his speech: he is ashamed of being someone who sells drugs, ashamed of not being able to support his family and make them proud. The mystifying dualism of this shame is that it is at once an isolating, intimately intra-psychic phenomenon seeking concealment, yet remains deeply embedded in a visual and public interpersonal space where the self is violently and unexpectedly exposed to the critical gaze of the Other (29). The source of shame can therefore never be completely in the self or in the Other, but is a rupture of what Kaufman (30) calls the “interpersonal bridge” (p. 22) binding the two. It is a disconnection and consequent lack of social recognition which underlies this shame.

Armin comments on the act of self-immolation in relation to his negative evaluation of his own life and thwarted expectations of living a normal life, a “human” life. The feelings expressed seem to be the same: injustice, solitude, sadness, emptiness, uselessness, all worsened by a perceived lack of hope in a better future. Here, time is a circular, wordless nothing. His act is defined explicitly as communicative, a direct call for social recognition:

I did it so that people could be made aware, to stop despising people like me, to know that all people are equal.

Continuing the interview, he paused and stated, “I don’t know. Maybe I’m mad.” It is impossible to grasp the full meaning behind these words. At face value, Armin seems to be considering whether or not he is indeed suffering from a psychiatric disorder, as stated by the many professionals with whom he has been in contact in Switzerland. However, throughout the two interviews we conducted with him, we are left with the impression that, for the most part, he appears to be contesting this very idea. Indeed, his words seem to reflect a resistance to the fact that he is a psychiatric patient in the face of others telling him that he is “mad.”

Although he finds himself cast out of the networks of humanity—having lost his family, his friends, his cultural homeland, his work, he justifies his act as a will to communicate his situation and humanity to other human beings. For Stolorow (23), being plunged into such singularity and solitude may paradoxically bring about an enhancement of “resoluteness” (p. 45). In such a state, the individual returns from the publicness of “they” to a more authentic and steadfast sense of self and purpose, which may lead to authentic “Being-toward-death.” In his own words, “Yes, I went to death.” However, on analyzing his motives for committing the act, it is evident that it goes beyond a simple desire to commit suicide. The self-stated reasons for him having committed the act highlight both the sociopolitical conditions in which he finds himself, as well as an internal psychological state of despair. This echoes the work of Biggs (31), who highlights the paradox that, in many cases, the act serves as both an escape and a protest.

Based on this hypothesis, considering the act “as escape” would arguably be indicative of suicidal ideation possibly linked to psychological factors of depression including a sense of helplessness and despair—in his own words “having no will to live.” Armin continues this reflection on his “situation without future” in which he finds himself. Not only does he perceive a life without future, he perceives a life where “dogs are treated better than me” and he is no better than “a cup.” He sees himself without a future and, most significantly, he connects this state to the lack of relationship with a social Other—in other words, his relationship to the network of human society which places him in the position of less than human, outside of social recognition.

## Discussion

Torn from the communal fabric of being-in-time, trauma remains insulated from human dialogue (23) (p. 56).

Disruptions created by trauma are embedded within an intersubjective context wherein severe emotional pain cannot find a relational home in which to be held and integrated (23, 24). Trauma disrupts the intersection of the individual and their social context and related to safety, trust, independence, power, esteem, intimacy as well as spiritual and existential beliefs. These disruptions been theorized as representing a threat not only to one’s core sense of self, but furthermore a violation of self-understanding and worldviews to the extent that it disrupts attachment and interpersonal dialogue necessary for meaning making in the social world (14, 32, 33). Particularly in cases where trauma has been prolonged, “the survivor may be left with large chunks of endured experience with no meaning, creating disquieting gaps and discontinuities in the experience of one’s life history” (16). This was the case of Armin who sees “no future” for himself as a “worthless person.”

For the transformation of traumatic memories into semiotic forms which connects it through language to its rightful place in time, the elaboration needs to be socially situated and “intersubjectively acknowledged” (34). This is because social resources provide a time orientation, and, consequently, a self-continuity between past and future (35) necessary for the construction of a coherent narrative, and, ultimately, the reconstruction of the Self. From a dialogical perspective, the psychological processing of trauma cannot merely be internally homogenous but involve multiple voices, texts, interests and traditions embodied in each individuals own varied histories and in the artifacts and norms of the system—a source of trouble and of innovation (25). This can only take place in the context of “interlocution” or “addressivity”—a dialogue between the person’s inner world and the sociocultural context in which traumatic events are processed (14, 36). The critical issue here is that of the notion of reciprocity (37) inherent to social recognition (21). Thus, it is within this dialectal sphere between the internal and external, Self and Other, the personal and political where coherent narratives of the event may be formed as part of the process of healing. As such, the self-immolation of Armin may be seen as a communicative act.

## Concluding Remarks

The interplay between language, trauma, and the sociocultural context is a complex and mutually reinforcing one: traumatic events may overwhelm and even rupture the semiotic systems in language which connects the individual to the communicative and social resources necessary to its very regulation and healing, thus perpetuating a vicious cycle of isolation and disconnection. The process of migration may be in itself a traumatic experience wherein the Self risks being annihilated and negated through the systemic trauma associated with legal and social practices of exclusion (14). In this space, social bonds and connections are disrupted which themselves are, paradoxically, necessary for finding the language to make sense of traumatic experiences in the form of a coherent narrative. Considering this cycle of trauma and disruption, it is noteworthy that Armin’s history of violence began before he claimed asylum, as evidenced by his attempt to gorge out the eye of a judge in his country of origin. This serves as an important reminder of the complex context of trauma—which extends beyond an isolated event. Furthermore, it highlights the compounded, interdependent relationship between the individual (who in this case arrived in Europe with a prior history of violence) and the social, cultural, and political context.

In the apparent absence of social recognition, Armin found himself “a worthless human.” Cast outside of the containment of human plurality as a result of a myriad of political and social mechanisms of exclusions, we hypothesize that his act of self-immolation serves as both an escape and a protest, both a “relational striving” for “being-in-the-world,”—profoundly embedded in an intersubjectively constitutive context, and a “being-toward-death” (23). Is the act both a significant indicator of deep psychological distress and despair, as well as an attempt to restore a connection to the world of the living? We argue that the utterance is at once disruptive and engaging, destructive and constructive, a conflict and a collaboration, a death instinct toward destruction but a “destruction as the cause of coming into being” (38). To quote Armin himself, it is at once a “yes, I went to death” as well as an “I want to live like other people.”

When he placed himself in the most public space of a Swiss town and set himself alight “to show that all men are equal,” it was an attempt to overcome trauma, social isolation, and lack of social recognition, a co-constructed inquiry to begin to try and put symbolic expression to experience (39). A “social interaction in its own right” (40), it represents an attempt to restore interaction. As such, it is a way of metaphorically construing and narrating experience; a compelling narrative enjoining others to take action (41). This is an utterance, a communicative act with the consequent potential to promote agency and civic engagement (42) which demands a response from the addressee, the Other. It demands and forces social recognition.

The brutality of such acts leave the public with little choice but to be disrupted and engaged as an “addressee”—whether voluntarily or not. In such moments, “the public sphere can no longer turn a blind eye to its privileged bodies” [Habermas, as cited in Ref. (43)], “the audience is not allowed to simply demonstrate ‘distant compassion’ but rather they are encouraged to engage and self-reflect about local injustices and activism within their own vicinities” (4) (p. 100). This self-immolation was a powerful communicative act that utilized self-inflicted violence as a means of forcing social recognition, both a personal and political action.

## Ethics Statement

This study was carried out in accordance with the recommendations of the Ethics Committee of the University of Neuchatel with written informed consent from all subjects in accordance with the Declaration of Helsinki. The protocol was approved by the Ethics Committee of Neuchatel.

## Author Contributions

The authors conducted fieldwork, conceptualized and wrote the paper together. GW is the first author, and LK is the second author and corresponding author.

## Conflict of Interest Statement

The authors declare that the research was conducted in the absence of any commercial or financial relationships that could be construed as a potential conflict of interest.

## Footnotes

^2^http://www.unhcr.org/figures-at-a-glance.html (Accessed: March 23, 2018).

^3^https://data.worldbank.org/indicator/SM.POP.REFG (Accessed: March 23, 2018).

^4^http://www.asylumineurope.org/reports/country/switzerland/statistics (Accessed: March 23, 2018).

^5^In the translation from French into English, the words may be translated either as “I surrendered to death” or “I went to death.”
